# A TRANSDISCIPLINARY TEAM APPROACH TO TECHNOLOGY DEVELOPMENT TO SUPPORT HEALTH AND WELL-BEING

**DOI:** 10.1093/geroni/igac059.1226

**Published:** 2022-12-20

**Authors:** Renato Ferreira Leitao Azevedo, Jennifer Nicholas, Jeannie Lee, Kathleen Insel, Wendy Rogers

**Affiliations:** University of Illinois Urbana-Champaign (UIUC), Urbana Champaign, Illinois, United States; The University of Arizona, Tucson, Arizona, United States; University of Arizona, Tucson, Arizona, United States; University of Arizona, Tucson, Arizona, United States; University of Illinois Urbana Champaign, Champaign, Illinois, United States

## Abstract

Diverse disciplinary and experiential knowledge is required to understand the challenges and develop workable solutions to support the health and wellbeing of a rapidly aging population. We are developing a theory-based, digital therapeutic for self-management of hypertension medication adherence for older adults; namely, the Medication Education, Decision Support, Reminding, and Monitoring (MEDSReM) system. This development process has necessitated a transdisciplinary approach with members co-learning and working collaboratively to advance the MEDSReM system. Our group includes complementary expertise in pharmacy, nursing, human factors, cognitive aging, gerontology, and technology development. We describe the challenges of transdisciplinary teamwork, our collaborative processes, and strategies that led to successful integration of expertise in advancing MEDSReM. Lastly, we discuss useful approaches to technology conceptualization, and digital therapeutic development and implementation, as well as lessons learned in effective communication and coordination among the diverse team members to ensure that the MEDSReM goals are achieved.

